# Peptide-Conjugated Phosphorodiamidate Morpholino Oligomers Retain Activity against Multidrug-Resistant Pseudomonas aeruginosa *In Vitro* and *In Vivo*

**DOI:** 10.1128/mBio.02411-20

**Published:** 2021-01-12

**Authors:** Dina A. Moustafa, Ashley W. Wu, Danniel Zamora, Seth M. Daly, Carolyn R. Sturge, Christine Pybus, Bruce L. Geller, Joanna B. Goldberg, David E. Greenberg

**Affiliations:** a Department of Pediatrics, Division of Pulmonary, Allergy and Immunology, Cystic Fibrosis, and Sleep, Emory University School of Medicine, Atlanta, Georgia, USA; b Emory+Children’s Center for Cystic Fibrosis and Airway Disease Research, Emory University School of Medicine, Atlanta, Georgia, USA; c Department of Internal Medicine, University of Texas Southwestern Medical Center, Dallas, Texas, USA; d Department of Microbiology, Oregon State University, Corvallis, Oregon, USA; e Department of Microbiology, University of Texas Southwestern Medical Center, Dallas, Texas, USA; Louis Stokes Veterans Affairs Medical Center

**Keywords:** *Pseudomonas aeruginosa*, antisense, PPMO, experimental therapeutics

## Abstract

Most antimicrobials currently in the clinical pipeline are modifications of existing classes of antibiotics and are considered short-term solutions due to the emergence of resistance. Pseudomonas aeruginosa represents a major challenge for new antimicrobial drug discovery due to its versatile lifestyle, ability to develop resistance to most antibiotic classes, and capacity to form robust biofilms on surfaces and in certain hosts such as those living with cystic fibrosis (CF). A precision antibiotic approach to treating *Pseudomonas* could be achieved with an antisense method, specifically by using peptide-conjugated phosphorodiamidate morpholino oligomers (PPMOs). Here, we demonstrate that PPMOs targeting *acpP* (acyl carrier protein), *lpxC* (UDP-(3-*O*-acyl)-*N*-acetylglucosamine deacetylase), and *rpsJ* (30S ribosomal protein S10) inhibited the *in vitro* growth of several multidrug-resistant clinical P. aeruginosa isolates at levels equivalent to those that were effective against sensitive strains. Lead PPMOs reduced established pseudomonal biofilms alone or in combination with tobramycin or piperacillin-tazobactam. Lead PPMO dosing alone or combined with tobramycin in an acute pneumonia model reduced lung bacterial burden in treated mice at 24 h and reduced morbidity up to 5 days postinfection. PPMOs reduced bacterial burden of extensively drug-resistant P. aeruginosa in the same model and resulted in superior survival compared to conventional antibiotics. These data suggest that lead PPMOs alone or in combination with clinically relevant antibiotics represent a promising therapeutic approach for combating P. aeruginosa infections.

## Abstract (precis)

Numerous Gram-negative bacteria are becoming increasingly resistant to multiple, if not all, classes of existing antibiotics. Multidrug-resistant Pseudomonas aeruginosa bacteria are a major cause of health care-associated infections in a variety of clinical settings, endangering patients who are immunocompromised or those who suffer from chronic infections, such as people with cystic fibrosis (CF).

## INTRODUCTION

Pseudomonas aeruginosa is among the most virulent opportunistic pathogens and continues to be recognized as a serious threat by the CDC (1). P. aeruginosa causes significant infection in immunocompromised hospitalized patients. This includes the development of chronic respiratory infection in patients with cystic fibrosis (CF) and non-CF bronchiectasis, which can lead to deteriorating lung function as well as increased morbidity and mortality (2). In addition to intrinsic and extrinsic drug resistance, P. aeruginosa has the ability to form robust biofilms, a pathogenic mechanism that makes treatment with small molecule antibiotics difficult (3).

With rapidly increasing rates in the emergence of multidrug-resistant (MDR) organisms, new antibacterial drug approaches are urgently needed. Antisense antibiotics, specifically peptide-conjugated phosphohorodiamidate morpholino oligomers (PPMOs), could be an innovative therapeutic strategy. The oligomer portion of the PPMO is designed to specifically bind to mRNA and prevent translation of the target protein of interest. The cell-penetrating peptide of the PPMO enhances cellular entry across Gram-negative membranes (4). PPMOs can target a variety of essential bacterial genes, including *acpP* (acyl carrier protein), *lpxC* (UDP-(3-*O*-acyl)-*N*-acetylglucosamine deacetylase), and *rpsJ* (30S ribosomal protein S10), and have inhibited the growth of multiple, clinically significant Gram-negative bacteria, including Acinetobacter baumannii (5), members of the Burkholderia cepacia complex (Bcc) (6, 7), Escherichia coli (8–12), and Salmonella enterica (13), *in vitro* and *in vivo*. We previously demonstrated that PPMOs can inhibit P. aeruginosa growth alone or in synergy with multiple clinically relevant antibiotics and reduce existing biofilm and bacterial lung burden in a fixed endpoint experiment in a P. aeruginosa acute pneumonia model (14). In this study, we expand upon our initial findings and determine that our lead *Pseudomonas* PPMOs can provide a survival benefit *in vivo* when delivered systemically or in a targeted fashion. In addition, we tested PPMOs against MDR and extensively drug-resistant (XDR) P. aeruginosa strains and attempt to enhance the activity of traditional antibiotics in the setting of biofilm.

## RESULTS

### PPMOs are effective against multi- (MDR) and extensively drug-resistant (XDR) P. aeruginosa.

We previously demonstrated that peptide-conjugated PMOs were effective at inhibiting the growth of several P. aeruginosa strains *in vitro* and this effect was further enhanced up to eightfold in the presence of subinhibitory concentrations of polymyxins or the cyclic decapeptide polymyxin B nonapeptide (PMBN) when cultured in cation-limiting media (14). In minimal media, our current lead PPMOs (targeting *acpP*, *lpxC*, or *rpsJ*) retained activity against both MDR and XDR clinical strains (Table 1). For any given PPMO, potency was similar regardless of the mechanism of resistance in that strain. For example, the MIC for AcpP-RXR(5′) (where R is arginine, X is 6-aminohexanoic acid, 5′ is peptide conjugation site; full PMO and peptide sequences are listed in Table S2 in the supplemental material) was 0.5 µM in strain PAO1, 0.25 µM in MB447, an XDR bloodstream isolate with a porin mutation (OprD truncation), and 0.5 to 1 µM in strains with various metallo-β-lactamase enzymes (MB580A, N7011285, and N3042857). Both cell-penetrating peptides ((RXR)_4_XB and (R_6_G); B is β-alanine and G is glycine) showed activity; however, PMOs conjugated to (RXR)_4_XB were more potent *in vitro* with MIC_75_ values of 0.5 to 16 µM compared to 4 to >32 µM with R_6_G conjugation (Table 1). In addition, the site of peptide conjugation to the PMO (5′ versus 3′) did not have a great impact on *in vitro* activity in these tests (Table 1). Preliminary cellular toxicity assays in THP1 monocytes and A549 lung epithelial cells for select PPMOs demonstrated cellular survival ranging from 90% to 100% after 48 h of incubation (see Fig. S1 in the supplemental material).

**TABLE 1:**
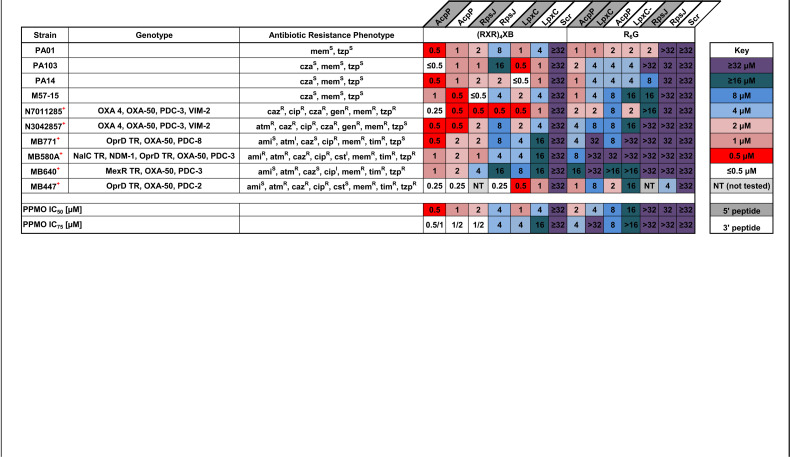
Heatmap of P. aeruginosa growth inhibition by PPMOs^*a*^

a Heatmap of PPMO MICs of P. aeruginosa strains grown in MOPS. A red + superscript in the Strain column indicates a multidrug-resistant strain. Scr (scrambled) PPMO was tested with corresponding peptide conjugation. Abbreviations: TR, truncation; caz, ceftazidime; cza, ceftazidime-avibactam; mem, meropenem; tzp, pipercillin-tazobactam; ami, amikacin; atm, aztreonam; cip, ciprofloxacin; cst, colistin; tim, ticarcillin-clavulanate; S, sensitive; I, intermediate; R, resistant.

10.1128/mBio.02411-20.1 FIG S1 PPMOs display modest cytotoxicity to A549 and THP-1 cells. Human A549 lung epithelial cells and THP-1 human monocyte cells (ATTC, Manassas, VA) were grown to about 1/8 confluency as described. PPMOs were added to a final concentration of 10 µM. After 48 h, cells were removed by brief trypsinization and mixed with trypan blue. Viability was quantified by microscopic examination and enumeration of dye-excluded cells/total cells. Results are presented as a percentage of cell survival compared to control at *T* = 0 before PPMO treatment. Data are presented as means ± standard deviations (SD) (*n* = 6). Results summarize two independent experiments. Download FIG S1, TIF file, 0.08 MB. Copyright © 2021 Moustafa et al. 2021 Moustafa et al. This content is distributed under the terms of the Creative Commons Attribution 4.0 International license.

### PPMOs in combination with traditional antibiotics display enhanced activity and reduce established biofilms.

**FIG 1:**
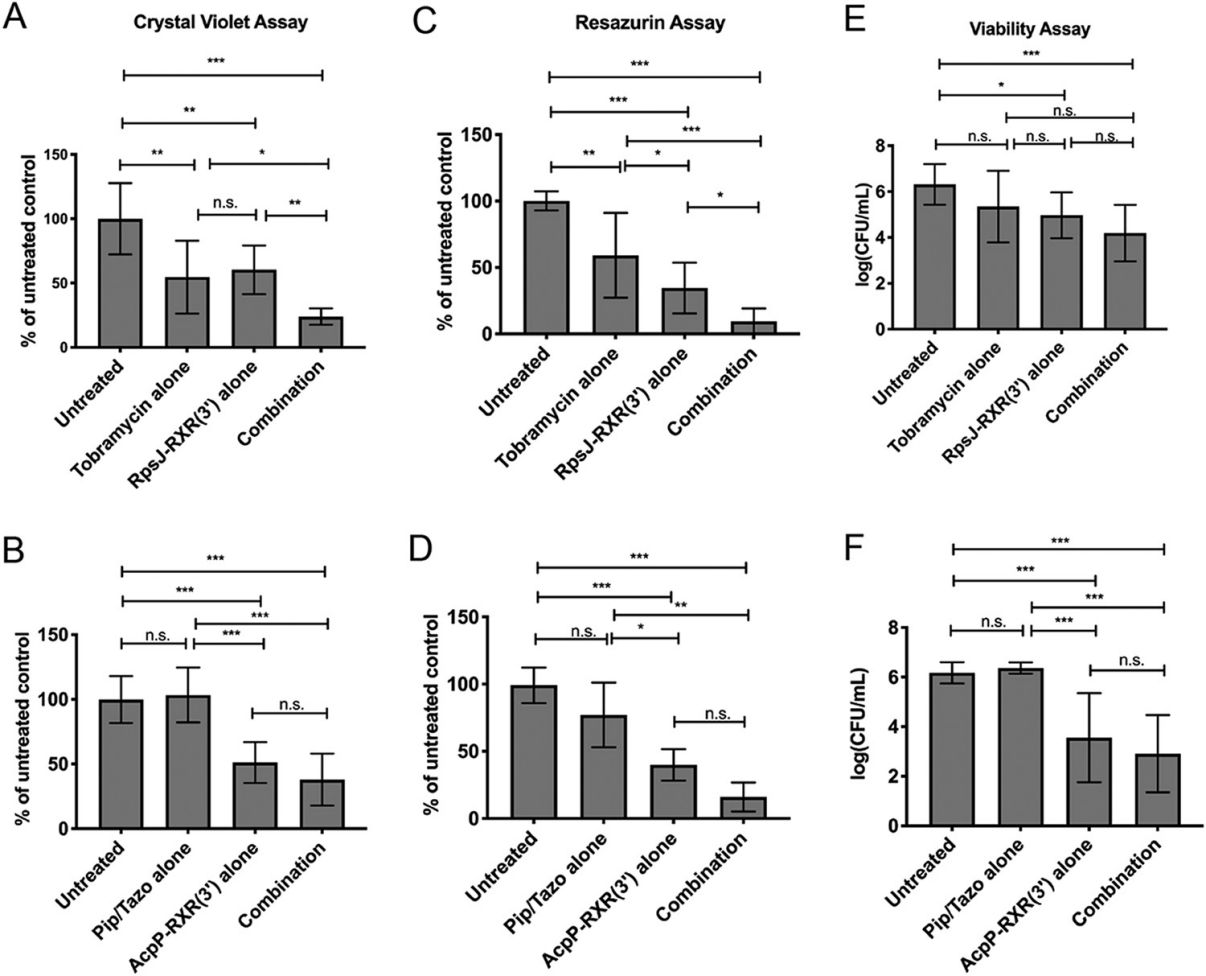
PPMOs in combination with traditional antibiotics display enhanced activity and reduce established P. aeruginosa strain PAO1 biofilm. PAO1 biofilms were established on MBEC pegs for 24 h and then treated either with 0.625 μg/ml tobramycin, 0.25 μg/ml piperacillin/tazobactam (Pip/Tazo), 2.5 μM AcpP-RXR(3′), 2.5 μM RpsJ-RXR(3′) or with a combination of PPMO and antibiotic at 24, 32, and 40 h. At 48 h, the MBEC pegs were processed by crystal violet staining (total biomass) (A and B), resazurin assay (viability and metabolism) (C and D), sonication and dilution for plate count CFU (viability) (E and F). Statistical differences were determined by one-way ANOVA and indicated as follows: ***, *P* < 0.0001; **, *P* < 0.001; *, *P* < 0.05; n.s., not significant.

We previously reported that treatment with PPMO alone was effective in both preventing and decreasing established P. aeruginosa biofilm *in vitro* (14). Here, we aimed to determine whether combining a PPMO with a traditional antibiotic (that also had minimal biofilm-inhibitory activity on its own) could enhance biofilm eradication compared to either drug alone. Biofilms were established in minimal biofilm eradication concentration (MBEC) plates by growing P. aeruginosa PAO1 at 37°C. At 24 h postinoculation, lids with established biofilm were moved to new 96-well plates containing fresh media with either antibiotic only (0.625 µg/ml tobramycin or 0.25 µg/ml piperacillin-tazobactam), PPMO only [0.25 µM either AcpP-RXR(3′) or RpsJ-RXR(3′)], or PPMO plus antibiotic as indicated. We hypothesized that the combination of antibiotics and PPMOs targeting a similar cellular process, such as bacterial cell wall (AcpP and piperacillin-tazobactam) or protein synthesis (RpsJ and tobramycin) would enhance biofilm eradication. We had previously demonstrated *in vitro* synergy with these combinations in planktonic culture (14). Multiple methods for measuring biofilm (crystal violet, resazurin, or CFU to detect viable bacteria) showed similar trends (Fig. 1). Treatment with RpsJ or AcpP alone resulted in significant reductions of biofilm mass and decreased cell viability by ∼50% compared to no treatment (Fig. 1). Piperacillin-tazobactam alone had no impact on established biofilm at the concentrations used, while tobramycin significantly reduced biofilm mass and cell viability by 50% compared to untreated cultures (Fig. 1). Overall, there was a trend toward significantly decreased biofilm with combination therapy compared to PPMO or antibiotic alone. These results correlated with confocal microscopy examination and demonstrated a decrease in both viable cells and biofilm thickness (Fig. S2). Although synergistic activity has not been confirmed, PPMO therapy and traditional antibiotics do not appear to be antagonistic in the biofilm setting *in vitro*.

10.1128/mBio.02411-20.2 FIG S2 PPMOs in combination with traditional antibiotics display enhanced activity and reduce established P. aeruginosa strain PAO1 biofilm. Live/Dead staining (red for propidium iodide, green for syto9) by spinning disk confocal microscopy (40×/1.3 NA). Download FIG S2, TIF file, 0.8 MB. Copyright © 2021 Moustafa et al. 2021 Moustafa et al. This content is distributed under the terms of the Creative Commons Attribution 4.0 International license.

### Delayed treatment with PPMOs reduce bacterial burden and increase survival in an acute pneumonia mouse model.

We previously demonstrated the therapeutic potential of 5′ conjugated (RXR)_4_XB PPMOs in an *in vivo* model of acute pneumonia. Treatment of BALB/c mice infected with P. aeruginosa 15 min postinfection with PPMOs targeting *acpP*, *lpxC*, or *rpsJ* significantly reduced the bacterial burden in the lungs at 24 h by almost 3 log units (14).

Treatment with PPMOs shortly after infection does not precisely simulate the way this therapeutic approach would likely be used in a patient. Therefore, we aimed to determine whether PPMOs could provide a survival advantage when delivered in a delayed fashion. In this current study, we build upon these earlier results by expanding our *in vivo* testing to include new compounds with (RXR)_4_XB and R_6_G peptides conjugated to the PMO at the 5′ or 3′ site.

**FIG 2:**
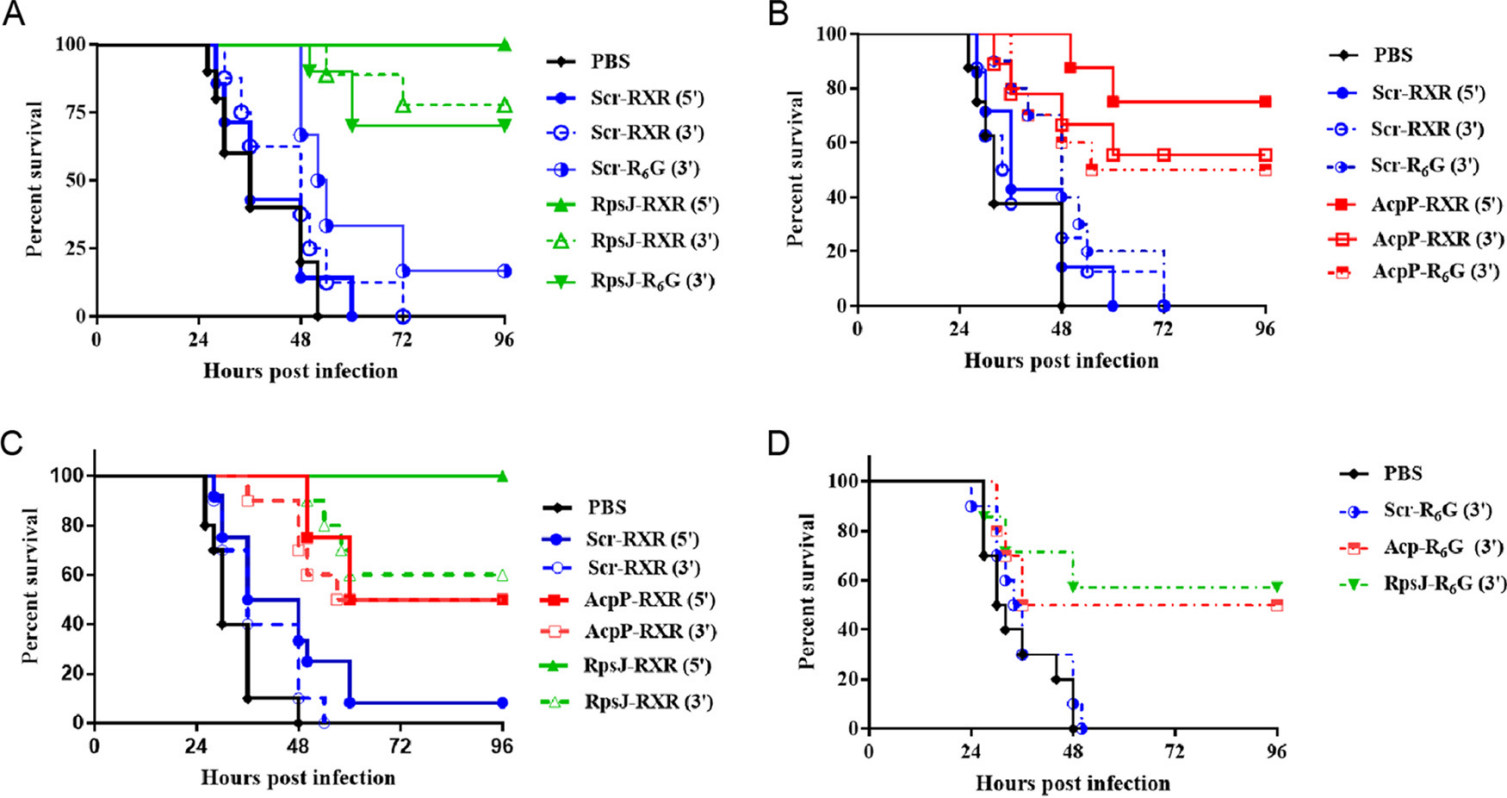
Multiple delayed intranasal and systemic treatment increases survival outcome in mice infected with P. aeruginosa PA103. Mice were infected intratracheally with P. aeruginosa PA103 or PA103 *P1-lux* (∼10^5^ CFU/mouse) and received intranasal treatments at 6, 12, and 24 h postinfection with RpsJ (A) or AcpP (B) or intraperitoneal (IP) treatments with RXR-conjugated (C) or R_6_G-conjugated (D) 5′ or 3′ conjugated peptides. Corresponding Scr and PBS were included. Each mouse received 15 mg/kg/treatment. Mice were monitored for 4 days postinfection. Results are represented by Kaplan-Meier survival curves and were analyzed by log rank test. For intranasal treatments, RpsJ versus PBS, *P *< 0.0001; RpsJ-RXR versus Scr-RXR, *P *< 0.0001; RpsJ-R_6_G versus Scr-R_6_G, *P *< 0.05. For AcpP compounds, log rank test, PBS versus all AcpP compounds, *P* < 0.01 to *P *< 0.0001; AcpP versus Scr, *P *< 0.05 to *P* < 0.001. For intraperitoneal treatments, AcpP-RXR and RpsJ-RXR versus PBS, *P *< 0.0001; RpsJ-RXR versus Scr-RXR, *P* < 0.0001; AcpP-RXR versus Scr-RXR, *P *< 0.01; all R_6_G-conjugated compounds versus PBS or Scr, *P *< 0.05.

Mice were infected via intratracheal instillation with a sublethal dose of P. aeruginosa PA103 and were treated intranasally with 15 mg of the indicated PPMOs or their corresponding scrambled (Scr) control per kg of body weight with a first dose at 6 h followed by repeat doses at 12 and 24 h postinfection. The mice were monitored for survival for up to 4 days postinfection. We first compared three different PPMOs targeting RpsJ (RpsJ-RXR(5′), RpsJ-RXR(3′), and RpsJ-R_6_G(3′)) to test the impact of either penetrating peptide and the attachment site of the peptide. Delayed treatment with any of the RpsJ PPMOs provided a statistically significant survival advantage compared to either Scr PPMO or phosphate-buffered saline (PBS) treatment (RpsJ versus PBS, *P* < 0.0001; RpsJ-RXR versus Scr-RXR, *P *< 0.0001; RpsJ-R6G versus Scr-R_6_G, *P *< 0.05) with survival ranging from 70% to 100% (Fig. 2A). As expected, all mice treated with PBS succumbed to infection by 48 h postinfection, and they were shortly followed by scrambled (Scr) PPMO-treated animals. Similarly, mice treated with any of the various AcpP PPMOs showed >50% survival (AcpP versus PBS, *P *< 0.01; AcpP versus Scr, *P* < 0.05) compared to controls without a significant difference based on peptide or peptide attachment (Fig. 2B). *In vivo* imaging utilizing IVIS imaging and a bioluminescent strain, PA103 *P1*-*lux*, correlated with the survival results and demonstrated clearance of bacteria in the lung at 24 h postinfection (Fig. S3).

10.1128/mBio.02411-20.3 FIG S3 Delayed intranasal treatments using various RXR-conjugated PPMOs reduces bacterial burden compared to the corresponding Scr PPMO. Real-time monitoring of mice infected with P. aeruginosa PA103 *P1*-*lux* and treated via the intranasal route at 6, 12, and 24 h postinfection with 15 mg/kg/treatment with RXR(3′)-conjugated PPMOs. Mice were imaged at 24 h using an IVIS Lumina LT III CCD camera. Imaging was performed from the ventral side of representative mice while the animals were under isoflurane anesthesia. The color bars indicate the intensity of the bioluminescence output, with red and blue denoting the high and low signals, respectively. Download FIG S3, TIF file, 0.5 MB. Copyright © 2021 Moustafa et al. 2021 Moustafa et al. This content is distributed under the terms of the Creative Commons Attribution 4.0 International license.

Although targeted delivery of PPMOs to the lung provided a survival advantage in this acute pneumonia model, we wanted to determine whether systemic delivery would also provide a survival advantage. Mice were infected as described above, and active or control PPMO was delivered intraperitoneally at a similar dose (15 mg/kg) and schedule (6, 12, and 24 h postinfection) as before. For RXR-PMO conjugates, the AcpP and RpsJ PPMOs provided a significant survival advantage over controls, ranging from 50 to 100% depending on the PPMO (RpsJ versus PBS or Scr, *P *< 0.0001; AcpP versus PBS or Scr, *P *< 0.01) (Fig. 2C). The peptide attachment site did not seem to make a great difference in these tests; however, RpsJ-RXR(5′) demonstrated 100% survival as was also the case for the intranasal delivery experiments. For R6G-PMO conjugates, the AcpP and RpsJ PPMOs also provided a significant survival advantage over controls, ranging from 50 to 60% (*P* < 0.05) (Fig. 2D). These studies demonstrate the ability of lead PPMOs to provide a survival benefit in an acute pneumonia model, and this benefit was retained with systemic delivery.

### PPMO treatments reduce P. aeruginosa burden in infected mice in a dose-dependent manner.

**FIG 3:**
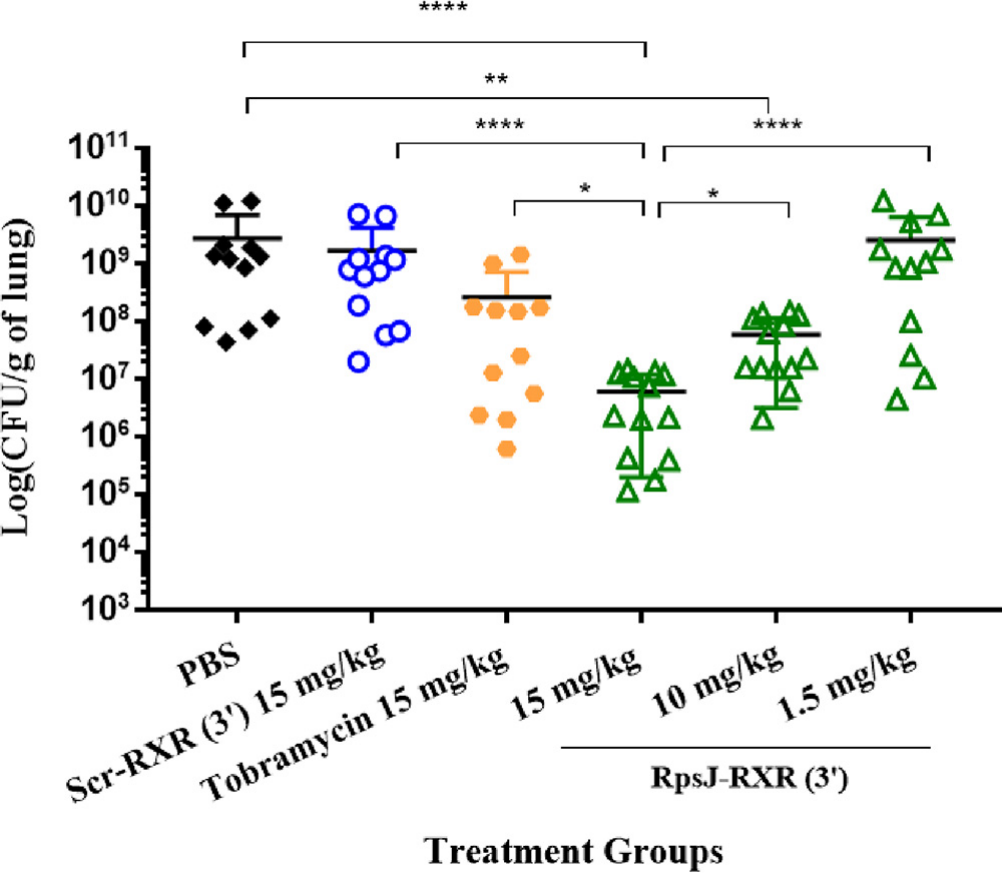
PPMO treatments reduce P. aeruginosa burden in infected mice in a dose-dependent manner. Mice were infected intratracheally with P. aeruginosa PA103 (∼10^5^ CFU/mouse) and treated intratracheally with various doses of RpsJ-RXR(3′), Scr-RXR(3′), tobramycin, or PBS at 15 min postinfection. Doses were the same for Scr and tobramycin (15 mg/kg/24 h). Doses for RpsJ were 15, 5, and 1.5 mg/kg/24 h. Mice were euthanized at 24 h postinfection, and lungs were aseptically collected and homogenized in 1 ml PBS. All samples were plated for viable CFU on *Pseudomonas* isolation agar. Each symbol represents the value for a single mouse. Error bars represent standard errors of the means (SEM). Statistical differences were determined by one-way ANOVA and indicated as follows: ****, *P* < 0.0001; **, *P* < 0.001; *, *P *< 0.05.

Although lead PPMOs could reduce lung burden in a fixed endpoint pneumonia model (14) and provide a survival benefit, we attempted to confirm whether PPMOs function in a dose-dependent manner. Mice were infected intratracheally with a sublethal dose of P. aeruginosa PA103, followed by a single intratracheal treatment 15 min postinfection with various doses (1.5 to 15 mg/kg) of RpsJ-RXR(3′) (Fig. 3). Control mice were infected and treated with either scrambled PPMO (15 mg/kg) or tobramycin (15 mg/kg), and bacterial burden was assessed at 24 h postinfection. Decreases in bacterial burden were dose dependent with a >2 log unit decrease with 15 mg/kg of RpsJ-RXR(3′) compared to either PBS or scrambled PPMO-treated mice. In addition, there was a statistically significant difference between the decrease (∼2 to 3 log unit decrease) in CFU seen with 15 mg/kg of the RpsJ PPMO compared to tobramycin (*P *< 0.05). The magnitude of CFU reduction was dose dependent with a smaller reduction in CFU compared to controls with reduced doses of RpsJ (1.5 and 10 mg/kg). Additionally, at a 10 mg/kg dose, RpsJ PPMO remained significant compared to PBS-treated mice (*P* < 0.01).

Alternative delivery routes for PPMOs include nebulization, and this approach was tested using the AcpP PPMO in the acute pneumonia model. Mice were infected as described above and were dosed with 3 mg/mouse (150 mg/kg) of the AcpP-RXR(5′) or corresponding scrambled PPMO at 6 and 18 h postinfection for a total of two treatment doses. Bacterial burden in treated mice was assessed at 24 h postinfection as previously described above. Our results show that mice receiving AcpP treatment showed ∼2 log unit reduction in the bacterial load (*P* < 0.01) compared to control mice treated with either scrambled PPMO or H_2_O (Fig. S4). These results indicate that PPMOs show dose-dependent decreases in lung burden in an acute pneumonia model and retain activity when aerosolized.

10.1128/mBio.02411-20.4 FIG S4 Aerosol treatment with RXR-conjugated AcpP reduces lung burden compared to the corresponding Scr PPMO. Mice were infected intratracheally with P. aeruginosa PA103 (∼10^5^ CFU/mouse) and exposed to an inhaled dose of 9 mg/ml/treatment of either AcpP or Scr PPMO at 6 and 18 h postinfection. Mice were euthanized at 24 h, and lung tissues were processed as previously described. All samples were plated for viable CFU on PIA. Each symbol represents the value for a single mouse. Error bars represent SEM. Statistical differences were determined using one-way ANOVA and indicated as follows: **, *P* < 0.01. Download FIG S4, TIF file, 0.07 MB. Copyright © 2021 Moustafa et al. 2021 Moustafa et al. This content is distributed under the terms of the Creative Commons Attribution 4.0 International license.

### PPMOs work in concert with traditional antibiotics to improve survival in an acute lung model of infection.

**FIG 4:**
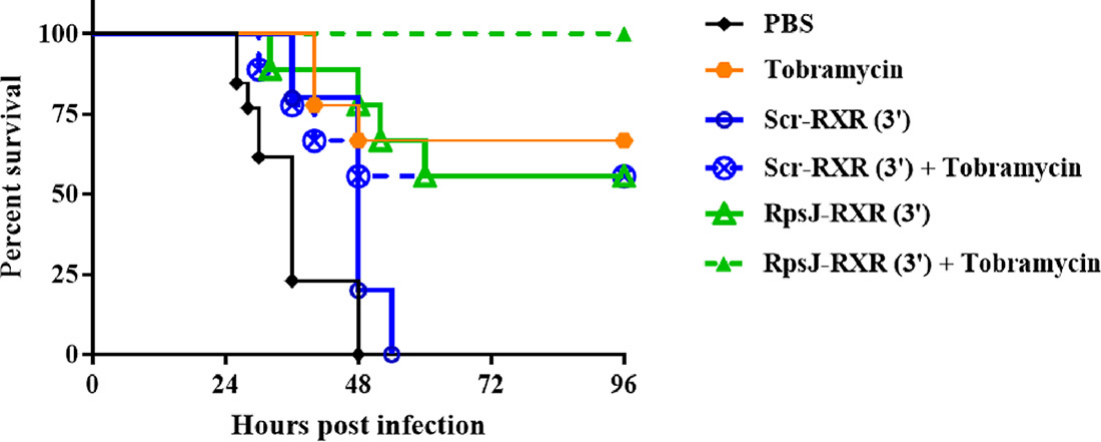
PPMOs work in concert with traditional antibiotics to improve survival in acute lung model of infection. Mice were infected intratracheally with P. aeruginosa PA103 (∼10^5^ CFU/mouse) and received intraperitoneal treatments at 6, 12, and 24 h postinfection with 15 mg/kg/treatment of RpsJ-RXR(3′) and the corresponding Scr alone or in combination with 20 mg/kg tobramycin. Mice were monitored for 4 days postinfection. Results are represented by Kaplan-Meier survival curves and were analyzed by log rank test (log rank test, RpsJ versus PBS, *P *< 0.001; RpsJ versus Scr, *P* < 0.05; RpsJ versus RpsJ plus tobramycin, *P *< 0.05; Scr versus Scr plus tobramycin, *P* = 0.1778. Median survival: PBS, 36 h; Scr, 48 h; RpsJ, tobramycin, and combination treatments, undefined).

Given that PPMOs displayed enhanced activity *in vitro* when combined with traditional antibiotics that target similar cellular pathways (14) in the biofilm setting, we tested whether combined therapy would provide an advantage *in vivo*. Mice were treated by intraperitoneal injection of RpsJ-RXR(3′) (15 mg/kg), tobramycin (15 mg/kg), scrambled PPMO (15 mg/kg) alone or in combination (scrambled PPMO plus tobramycin or RpsJ PPMO plus tobramycin) at 6, 12, and 24 h for a total of three treatments after intratracheal infection with a sublethal dose of P. aeruginosa PA103. Mice were monitored for up to 4 days postinfection. As expected, we found that mice treated with PBS or scrambled PPMO alone succumbed to infection by 48 h postinfection (Fig. 4). Mice treated with RpsJ-RXR(3′) or tobramycin had 50 to 60% increased survival compared to PBS or scrambled control, respectively. In contrast to single treatment, coadministration of RpsJ PPMO plus tobramycin resulted in 100% survival (RpsJ versus RpsJ plus tobramycin, *P* < 0.05). While these experiments do not prove *in vivo* synergy, it appears that this combination of a PPMO and small molecule antibiotic are not antagonistic and are at least additive *in vivo*.

### PPMOs reduce lung bacterial burden and provide a survival advantage in XDR P. aeruginosa.

**FIG 5:**
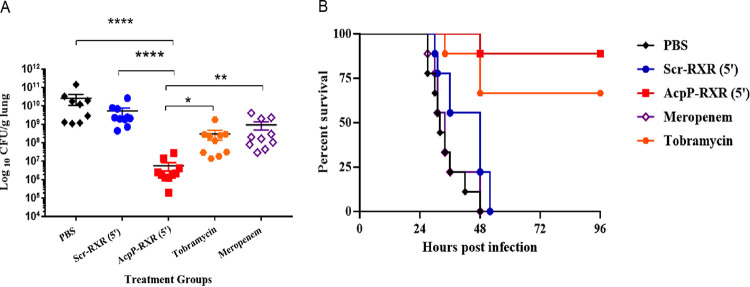
PPMOs provide a therapeutic advantage against XDR P. aeruginosa compared to traditional antibiotics. Mice were infected intratracheally with P. aeruginosa strain MB447 (∼10^7^ CFU/mouse). (A) Mice were treated intratracheally 15 min postinfection with 15 mg/kg/24 h of AcpP-RXR(5′) and the corresponding scrambled PPMO, tobramycin, and meropenem and were euthanized at 24 h, and lung tissues were processed as previously described. All samples were plated for viable CFU on PIA. Each symbol represents the value for a single mouse. Error bars represent SEM. Statistical differences were determined using one-way ANOVA and indicated as follows: ****, *P *< 0.0001; **, *P *< 0.01; *, *P* < 0.05. (B) Mice were treated intratracheally 15 min postinfection and intranasally at 6 h postinfection (a total of two treatments) with the same compounds and doses. Mice were monitored for 4 days postinfection. Results are represented by Kaplan-Meier survival curves and were analyzed by log rank test (log rank test, AcpP versus PBS or Scr or Meropenem, *P *< 0.0001. Median survival: PBS, 32 h; Scr, 48 h; meropenem, 34 h; AcpP and tobramycin, undefined). The MICs of strain MB447 against tobramycin and meropenem were 8 and 32 µM, respectively.

Carbapenems are frequently one of the last lines of defense antibiotics used in treating infections caused by P. aeruginosa. The emergence of isolates displaying an XDR phenotype, including carbapenem-resistant strains, has become increasingly prevalent. To test whether PPMOs retained *in vivo* activity in an XDR strain, we utilized the AcpP-RXR(5′) PPMO in the acute pneumonia model using XDR P. aeruginosa strain MB447, a carbapenem-resistant bloodstream isolate. Whole-genome sequencing revealed that this strain displayed extended-spectrum β-lactamase-related genes (PDC-2; OXA-50) and an OprD truncation (15). Despite resistance to most antibiotic classes, most PPMOs retained *in vitro* activity with MICs similar to those of drug-sensitive strains (Table 1). Mice were infected intratracheally followed by intratracheal treatment 15 min postinfection with either 15 mg/kg of AcpP-RXR(5′), scrambled control PPMO (Scr), PBS, 15 mg/kg of meropenem, or 15 mg/kg of tobramycin, and the bacterial burden was determined at 24 h postinfection. The AcpP PPMO resulted in an ∼3 log unit decrease in lung burden compared to PBS or scrambled control PPMO (*P *< 0.0001) (Fig. 5A). As expected, AcpP PPMO resulted in a significant reduction in CFU by ∼1 to 2 log units compared to either tobramycin or meropenem (AcpP PPMO versus tobramycin, *P *< 0.05; AcpP PPMO versus meropenem, *P *< 0.01). In a survival experiment, intratracheal treatment at 15 min postinfection followed by intranasal treatment at 24 h with the AcpP PPMO was superior to tobramycin or meropenem, with 90% of the AcpP group surviving at day 4 compared to 67% survival with tobramycin and 0% with meropenem (Fig. 5B), confirming that the AcpP PPMO retained *in vivo* superiority in this XDR P. aeruginosa lung infection.

## DISCUSSION

Treatment of P. aeruginosa infections has become increasingly difficult, and traditional antipseudomonal antibiotics are frequently ineffective due to various intrinsic resistance mechanisms (16). In addition, acquisition of resistance genes via horizontal gene transfer further contributes to the emergence of MDR and XDR phenotypes, leading to limited therapeutic options and potentially untreatable P. aeruginosa infections (17). The ability of P. aeruginosa to form robust biofilms further complicates these infections. Biofilm structures provide bacterial communities enormous advantages by evading host defenses and increasing tolerance to antimicrobial therapy through numerous adaptive mechanisms, including low metabolic activity and hypoxic conditions (18). The inability of antibiotics to eradicate biofilms has been classically attributed to the decreased and restricted ability of these compounds to penetrate the established biofilm due to the mechanical and chemical properties of the extracellular matrix (16). Moreover, experimental evidence demonstrates that P. aeruginosa biofilm-associated cells are significantly more resistant to antibiotics than their planktonic counterparts (18).

As a result, new approaches for developing antibiotics with activity against *Pseudomonas* are desperately needed that do not rely on modifications of existing scaffolds of traditional small molecule drugs. One approach is to utilize antisense molecules, and here we describe the use of phosphorodiamidate morpholino oligomers (PMOs) to target *Pseudomonas* in a gene-specific, species-specific way. The conjugation of PMOs to various peptides allows for efficient entry of the antisense molecule through the Gram-negative cell membrane through mechanisms that are not completely understood. PPMOs are thought to act through translation inhibition, as previous studies using PPMOs against nonessential genes show reduction in the protein product that is targeted (19). We have utilized this approach to target nonessential resistance genes such as NDM-1, MCR-1, or drug efflux components (8, 11, 20) as well as essential genes in medically important Gram-negative pathogens (5, 6, 21). Our prior efforts demonstrated that PPMOs have activity in P. aeruginosa *in vitro* and in a fixed endpoint *in vivo* acute pneumonia model. In the current study, we build upon these initial observations in important ways. First, we demonstrate that our lead PPMOs targeting *acpP*, *rpsJ*, and *lpxC* retain activity against MDR and XDR strains. This is critically important, as new antibiotics must demonstrate activity in these types of isolates. Strikingly, similar MICs were achieved in isolates regardless of their specific antibiotic mechanism of resistance. For example, the AcpP-RXR(5′) PPMO had an MIC of 0.5 µM against the laboratory strain PAO1, 0.25 to 1 µM against strains with metallo-β-lactamases, <0.5 µM against a strain with a porin mutation, and 1 µM in strains with multiple identified resistance determinants, including mutations in regulator genes associated with a hypereffluxer state (22). These results do not imply that resistance will not occur with an antisense approach but that the mechanism by which PPMOs enter the cell and function are not subject to classic mechanisms of resistance. Ongoing studies involve determining the frequency and mechanism of resistance of PPMOs in *Pseudomonas*. In addition, the activity of PPMOs in the biofilm setting provide an important advantage over the use of small molecule antibiotics. Although PPMOs are high molecular weight molecules, their activity in biofilm could be due to the cationic peptide moieties that lead to disruption of the negatively charged biofilm structure that allow for adequate penetration and access to the bacteria (23, 24). While we previously demonstrated that lead PPMOs alone could reduce existing biofilm, here we asked whether PPMOs combined with traditional antibiotics could lead to enhanced biofilm eradication over either compound alone. This appears to be the case, and while our PPMOs are being developing as single agents against *Pseudomonas*, combination therapy could certainly be considered. Although the PPMOs increased survival and reduced bacterial burden *in vivo*, further investigation will be necessary to conclude whether these effects were partially due to penetration of biofilm *in vivo.*

Lead PPMOs demonstrated a survival advantage in an acute pneumonia model, an aggressive infection model where control treated mice die by 48 h. Despite a delay in the first dose of PPMO, survival ranged from 50 to 100% depending on the PPMO and route of administration. Treatment with PPMOs directly to lung (including intranasal administration and nebulization) as well as systemic delivery showed activity. It is likely that PPMOs could be developed for systemic infections or targeted delivery, particularly in certain patient populations, such as those with CF. Further studies are needed to determine the optimal lead PPMO, as there was not a clear compound that was superior in these studies, although there was a slight trend toward improved *in vitro* and *in vivo* activity with 5′ peptide conjugates. Although the (RXR)_4_XB peptide showed better *in vitro* activity, this did not necessarily translate to the *in vivo* experiments, as both (RXR)_4_XB and R_6_G performed similarly well; however, the mechanisms behind these differences are unclear. Future pharmacokinetic (PK)/pharmacodynamic (PD) studies will be critical to determining optimal dosing of different peptide conjugates. In addition, while *in vitro* toxicity studies are encouraging, *in vivo* toxicity studies are under way that will be important in determining a final gene target and peptide combination to move into advanced preclinical development.

Successful development of *Pseudomonas* PPMOs could usher in a new paradigm for antibiotic development, specifically utilizing an approach that focuses on pathogen-specific therapies. While there are numerous variables that have fueled the rapid increase in antibiotic resistance, the use of broad-spectrum agents and their impact on the microbiome are certainly factors contributing to this crisis. This makes pathogen-specific therapeutics an attractive alternative, and indeed, there are multiple efforts under way developing other narrow-spectrum approaches, including phage or antibody therapeutics (25). There are currently no antibiotics on the market targeting *acpP* or *rpsJ*, making these potential new targets for development. LpxC inhibitors have been developed and are in various stages of preclinical development (26, 27). Here, we have shown that this platform technology can be a tool to confirm existing antibacterial targets. However, in this case, the tool (PPMOs) could be the drug itself. Importantly, a PMO is already FDA approved for the treatment of Duchenne muscular dystrophy (DMD), illustrating that extensive safety data exist in humans for the core molecule. In addition, a PPMO is currently in clinical trials for DMD as well. As the use of rapid diagnostics and whole-genome sequencing is becoming more common, the use of species-specific therapeutics becomes an increasingly attractive strategy. PPMOs could be one such approach for P. aeruginosa, and the *in vitro* and *in vivo* activity demonstrated in MDR strains supports continued development of these compounds.

## MATERIALS AND METHODS

### Bacterial strains.

All P. aeruginosa strains used in this study were either obtained from the American Type Culture Collection (Manassas, VA) or were clinical isolates (kindly provided by Sam Shelburne at MD Anderson Cancer Center, Houston, TX). Constitutively bioluminescent P. aeruginosa PA103 strain was constructed by immobilizing the plasmid pUC18miniTn7T-*lux*-Tp into the wild-type PA103 strain to generate PA103 *P1-lux* according to the method previously described (28). The strains used in this study are listed in Table S1 in the supplemental material.

10.1128/mBio.02411-20.5 TABLE S1 P. aeruginosa strain isolates used in this study. Download Table S1, DOCX file, 0.01 MB. Copyright © 2021 Moustafa et al. 2021 Moustafa et al. This content is distributed under the terms of the Creative Commons Attribution 4.0 International license.

### PPMO design and synthesis.

PMO sequences were designed as previously described (14). All compounds were synthesized and conjugated to peptides by Sarepta Therapeutics, Inc. (Cambridge, MA). PPMO gene targets, sequences, peptides, and peptide conjugation sites (5′ versus 3′) are indicated in Table S2. Scrambled (Scr) PPMO controls are random 11-mer sequences conjugated to the same peptides in the same orientation as that of the active PPMO. All compounds are delivered after manufacture as lyophilates and then solubilized in water for *in vitro* and *in vivo* experiments.

10.1128/mBio.02411-20.6 TABLE S2 Lead PPMOs used. Compounds are listed in alphabetical order. For the location relative to the start site, we defined “A” of ATG as +1. Abbreviations are as follows: R, arginine; G, glycine; X, 6-aminohexanoic acid (aminocaproic acid); B, β-alanine (for conjugation); TEG, triethylene glycol; Scr, scrambled PPMO. Download Table S2, DOCX file, 0.02 MB. Copyright © 2021 Moustafa et al. 2021 Moustafa et al. This content is distributed under the terms of the Creative Commons Attribution 4.0 International license.

### Bacterial susceptibility testing.

MIC assays of PPMOs were determined according to the Clinical and Laboratory Standards Institute (CLSI) broth microdilution method (29), using the method previously described (14). The assays were performed in 96-well tissue culture plates (Thermo Fisher Scientific, Waltham, MA). All reagents were analytical grade and were obtained from Sigma-Aldrich (St. Louis, MO), Acros Organics (Geel, Belgium), UT Southwestern Campus Pharmacy, or Thermo Fisher Scientific (Waltham, MA). Morpholinopropanesulfonic acid (MOPS) minimal medium was prepared as previously described (30, 31).

### Cell culture cytotoxicity assay.

Human lung epithelial cells A549 (ATCC) were grown in Ham’s F-12K medium supplemented with glutamine and 10% heat-inactivated fetal bovine serum (FBS). THP-1 human monocyte cells (ATCC) were grown in RPMI supplemented with glutamine, 25 mM HEPES, and 10% heat-inactivated FBS. Cells were grown in 24-well plates overnight at 37°C and 5% CO_2_. PPMOs were added to a final concentration of 10 µM. At time (*T*) = 0, 24, and 48 h, cytotoxicity was measured by counting viable cells using trypan blue exclusion assay (21, 32).

### Biofilm assays.

Biofilm breakdown assays were performed in 96-well minimum biofilm eradication concentration (MBEC) plates (Innovotech, Inc., Edmonton, Alberta, Canada). P. aeruginosa PAO1 was inoculated at 5 × 10^5^ CFU/ml in 150 µl of Mueller-Hinton II (MHII) broth per well, followed by incubation at 37°C with shaking at 110 rpm. At 24 h postinoculation, plate lids containing pegs with the established biofilm were moved to a new 96-well plate with fresh MHII medium treated with piperacillin-tazobactam (Fresenius Kabi-USA LLC), active PPMO, or a combination of PPMO plus antibiotic. The dosing was repeated at 32 and 40 h postinoculation for a total of three doses. Eight hours after the last dose, the MBEC lids with biofilm were processed by using a crystal violet assay for total biomass according to the method previously described (14).

### Viability assays.

The viability and metabolic state of biofilm-associated cells were determined using resazurin dye (17). Briefly, MBEC pegs were rinsed in phosphate-buffered saline (PBS) and transferred to a 96-well plate containing MHII with 100 μM resazurin and incubated at 37°C and 110 rpm for 6 h. This time point was previously optimized based on the time needed for the untreated control cells to reach maximum fluorescence. The plates were read at an excitation of 530 nm and emission of 590 nm on a Synergy BioTek plate reader. Viable CFU from the biofilms were also determined by rinsing MBEC pegs in PBS three times to remove planktonic bacteria, followed by cutting the peg off the base with a hot scalpel and placing it in a 14 ml Falcon tube containing 1 ml PBS. Tubes were sonicated in a room temperature water bath for 20 min, vortexed, serially diluted, and plated on sheep blood agar to enumerate the CFU.

### Spinning disk confocal microscopy.

MBEC pegs with established P. aeruginosa biofilms were rinsed in 150 mM NaCl, stained in a mixture of 3 μl Syto9, 3 μl propidium iodide, and 1 ml of 150 mM NaCl for 30 min in the dark, and then imaged on an Axiovert 200 M inverted microscope (Carl Zeiss, Thornwood, NJ) with an UltraVIEW ERS spinning disk confocal head (Perkin-Elmer, Waltham, MA) using a 40×/1.3 numerical aperture (NA) objective. Two channels were used to visualize live/dead staining: green (syto9, live) and red (propidium iodide, dead). Z-stacks had a spacing of 0.5 μm. Images were deconvoluted using Autoquant X3 software (Media Cybernetics, Inc., Rockville, MD) and rendered in IMARIS software (Bitplane USA, Concord, MA).

### Murine infection assays.

All animal procedures were conducted according to the guidelines of the Emory University Institutional Animal Care and Use Committee (IACUC), under approved protocol number DAR-2003421-042219BN. The study was carried out in strict accordance with established guidelines and policies at Emory University School of Medicine, and recommendations in the *Guide for Care and Use of Laboratory Animals* (33), as well as local, state, and federal laws. P. aeruginosa strains PA103, PA103 *P1*-*lux*, and MB447 were grown on *Pseudomonas* isolation agar (PIA) for 16 to 18 h at 37°C and suspended in PBS to an optical density at 600 nm (OD_600_) of 0.5, corresponding to ∼10^9^ CFU/ml. Inocula were adjusted spectrophotometrically to obtain the desired sublethal challenge dose in a volume of 25 μl. Six- to 8-week-old female BALB/c mice (Jackson Laboratories, Bar Harbor, ME) were anesthetized by intraperitoneal injection of 0.2 ml of a mixture of ketamine (6.7 mg/ml) and xylazine (1.3 mg/ml). Mice were infected by noninvasive intratracheal instillation of dilutions of P. aeruginosa strain as indicated (34).

### Delayed treatment experiments.

Mice were infected as described above and treated either intraperitoneally or intranasally with 15 mg/kg/treatment of PPMO or corresponding controls as indicated. Animals were treated at 6, 12, or 24 h postinfection, with a total of three doses and were monitored for survival up to 4 days postinfection.

For intranasal treatments, mice were infected with PA103 *P1*-*lux*, and colonization and localization of bioluminescent bacteria was monitored in real time using an IVIS Lumina LT III imaging system (PerkinElmer). Briefly, each group of mice was anesthetized with 3% isoflurane using an XGI-8 gas anesthesia system (Caliper Life Sciences), and imaged using medium binning, f/stop 1, subject height 1.5 cm. Images were acquired with up to 5-min exposures. Total photon emission from the ventral and dorsal sides of imaged mice were quantified using Living Image Software v4.0x (Xenogen Corp.). All correlations were done as average radiance of photons emitted per second, area, and steradian (p/s/cm^2^/sr) under the chosen experimental conditions.

### Dose response and fixed endpoint experiments.

Female BALB/c mice were infected as described above and treated intratracheally once at 15 min postinfection with various doses of PPMOs or antibiotics (tobramycin or meropenem) as indicated. Mice were euthanized at 24 h postinfection, and whole lungs were collected aseptically, weighed, and homogenized in 1 ml of PBS. Tissue homogenates were serially diluted and plated on Difco *Pseudomonas* isolation agar (PIA; BD Biosciences) for CFU determination.

### Aerosol treatment.

A small nose-only exposure chamber was used for inhalation delivery of PPMOs to mice. During experiments, the test particles were aerosolized using an Aeroneb Lab apparatus (Aerogen Inc., Galway, Ireland) connected to the multidosing animal chamber. The nebulizer was optimized to produce particles approximately 4 µm in diameter in a low velocity aerosol. Animals were individually placed into CH’47 tubes (CH Technologies, Westwood, NJ) and were positioned so that the mouse’s nose was at the inhalation point to ensure “nose only” delivery of the therapeutic compounds. Aerosolized active PPMO and corresponding scrambled compound were diluted to 9 mg/ml. Mice were treated at 6 and 18 h postinfection for a total of two doses. Mice were euthanized at 24 h postinfection, and the bacterial burden was evaluated as described above.

### Statistical analyses.

All analyses were performed using GraphPad Prism version 6 software. Results were analyzed using one-way analysis of variance (ANOVA) and were compared using the Kruskal-Wallis test for comparison of three groups or the Mann-Whitney *U* test for analysis of two groups. The results of survival studies were represented using Kaplan-Meier survival curves and were analyzed by the log rank test.
