# Supplementary figures and images for: Peptide-Conjugated Phosphorodiamidate Morpholino Oligomers Retain Activity against Multidrug-Resistant Pseudomonas aeruginosa In Vitro and In Vivo
Source: mBio. 2021 Jan 12;12(1):e02411-20. doi: 10.1128/mBio.02411-20 (PMC7844538; doi:10.1128/mBio.02411-20)

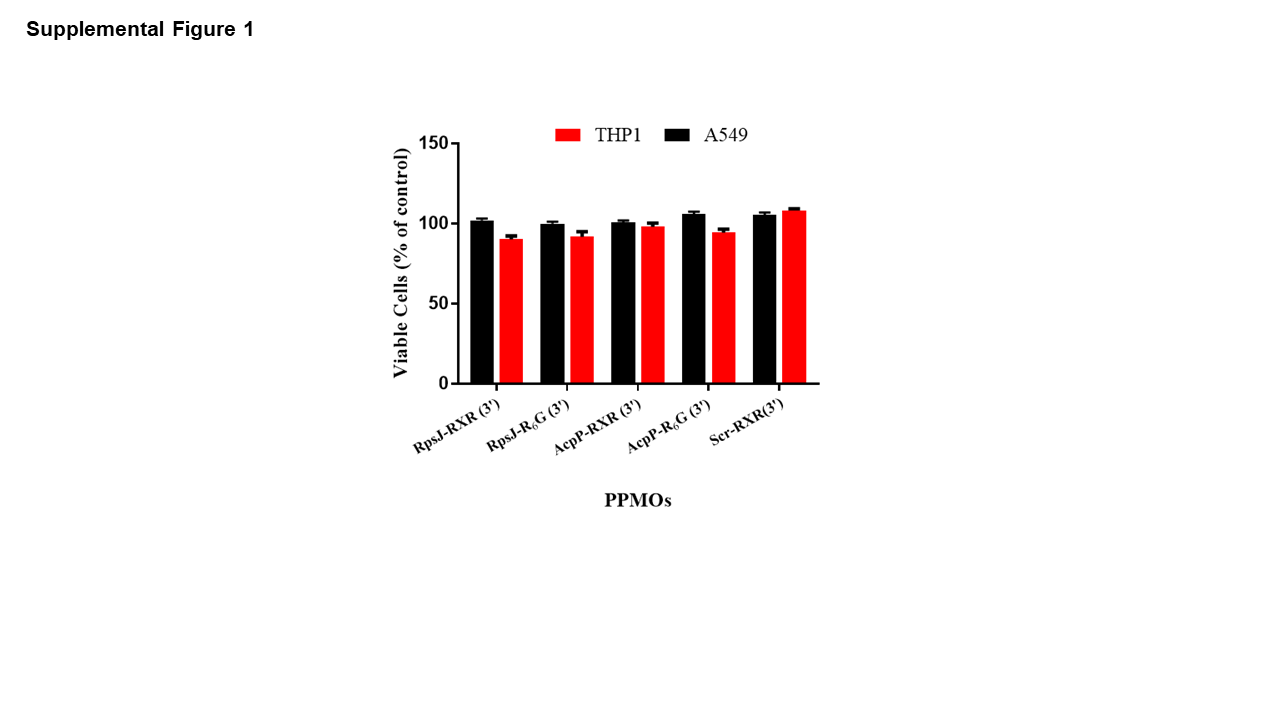

Supplement: FIG S1 [file mBio.02411-20-sf001.tif]

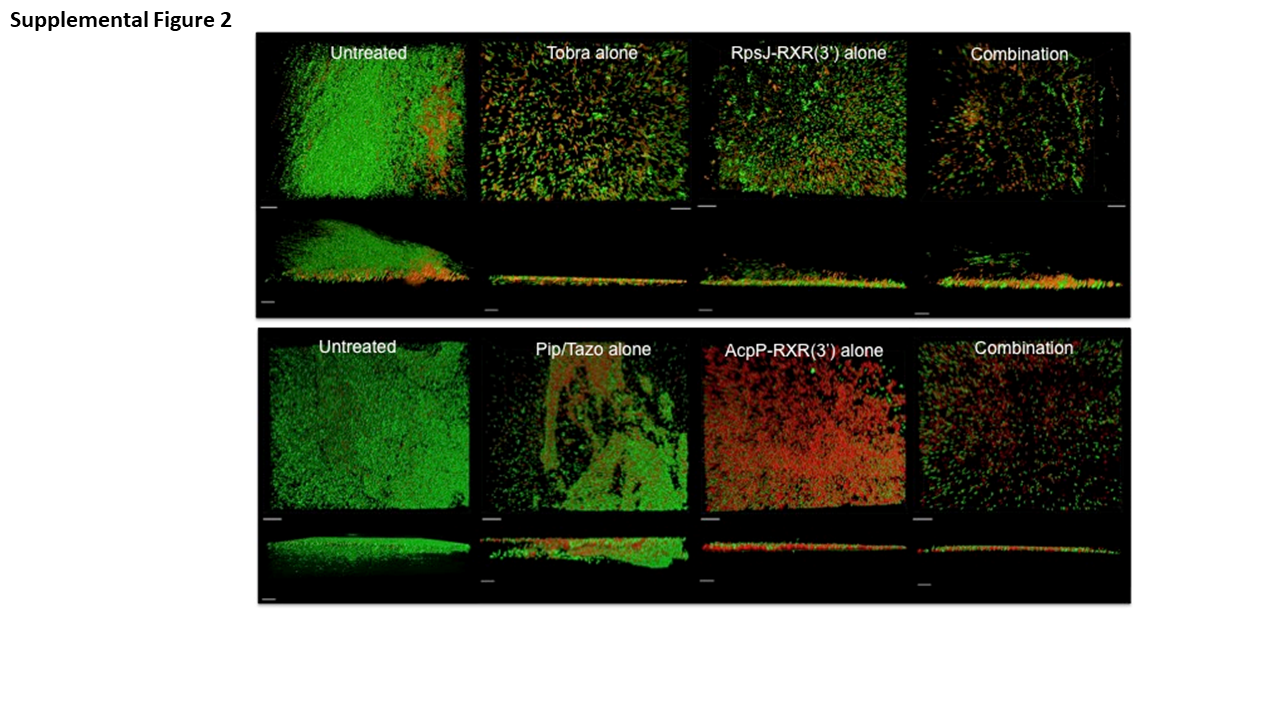

Supplement: FIG S2 [file mBio.02411-20-sf002.tif]

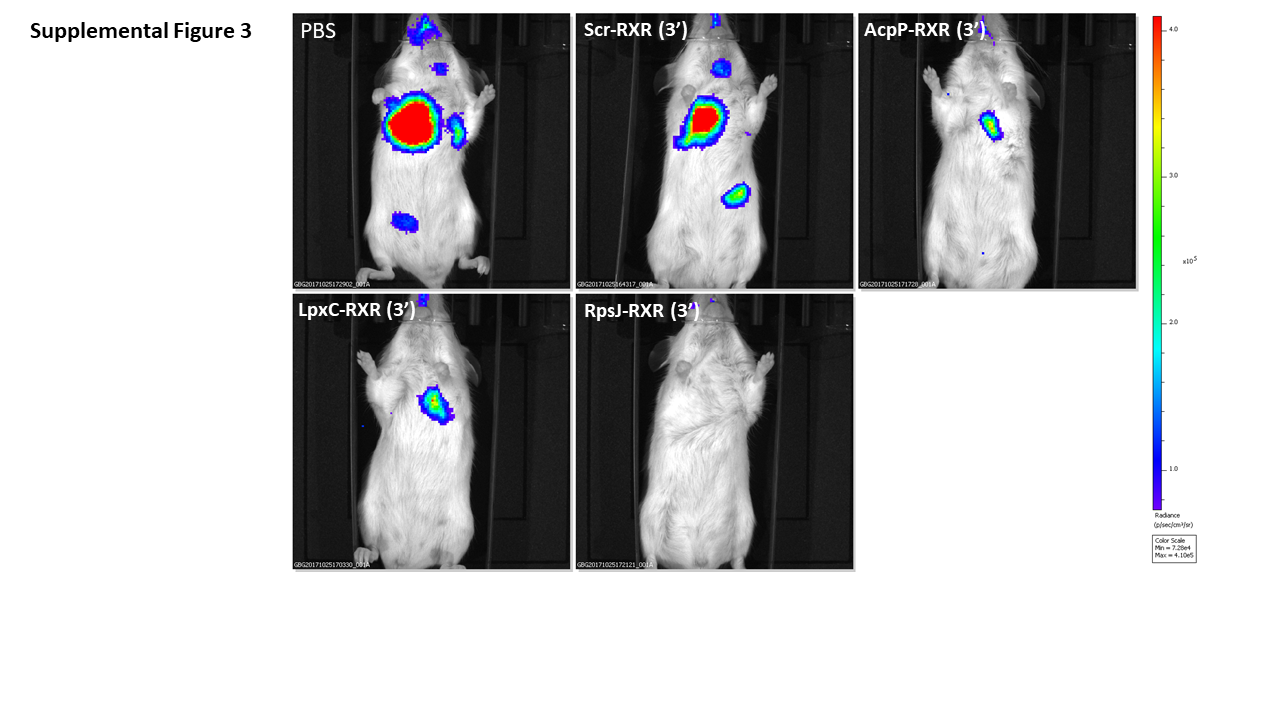

Supplement: FIG S3 [file mBio.02411-20-sf003.tif]

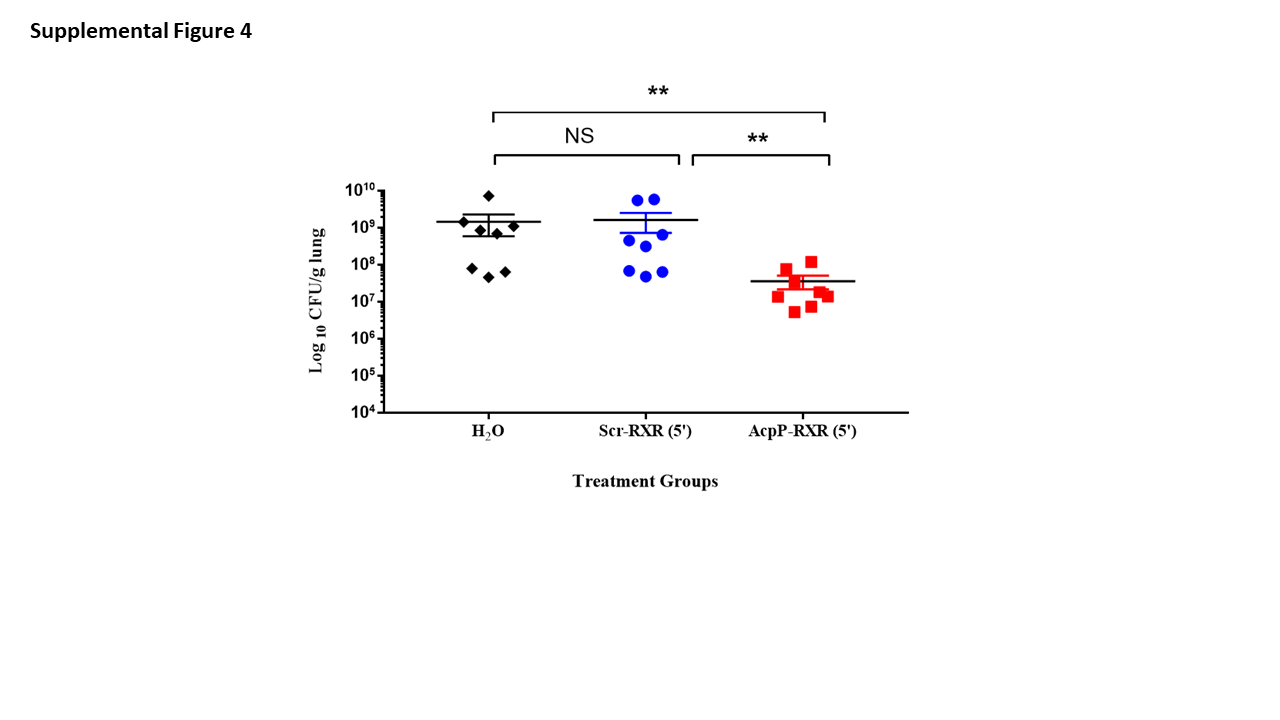

Supplement: FIG S4 [file mBio.02411-20-sf004.tif]
