# Supplementary material for: Peptide-Conjugated Phosphorodiamidate Morpholino Oligomers Retain Activity against Multidrug-Resistant Pseudomonas aeruginosa In Vitro and In Vivo
Source: mBio. 2021 Jan 12;12(1):e02411-20. doi: 10.1128/mBio.02411-20 (PMC7844538; doi:10.1128/mBio.02411-20)
Supplement: TABLE S1 [file mBio.02411-20-st001.docx]

**Supplemental Table 1. *P. aeruginosa* strain isolates used in this study**

| ***P. aeruginosa* strain** | **Source** |
| --- | --- |
| PAO1 | Infected wound (ATCC15692) |
| PA103 | Sputum (ATCC 29260) |
| PA14 | Burn wound infection |
| M57-15 | Mucoid CF isolate |
| N7011285 | Blood |
| N3042857 | Tracheal aspiration |
| MB771 | Blood |
| MB580A | Blood |
| MB640 | Blood |
| MB447 | Blood |

**Table S.1:** *P. aeruginosa* strain isolates used in this study
