# Supplementary material for: Peptide-Conjugated Phosphorodiamidate Morpholino Oligomers Retain Activity against Multidrug-Resistant Pseudomonas aeruginosa In Vitro and In Vivo
Source: mBio. 2021 Jan 12;12(1):e02411-20. doi: 10.1128/mBio.02411-20 (PMC7844538; doi:10.1128/mBio.02411-20)
Supplement: TABLE S2 [file mBio.02411-20-st002.docx]

**Supplemental Table 2: Lead PPMOs used.**

| **PPMO ID** | **Target gene** | **PPMO sequence 5’> 3’** | **DNA target** | **Location** | **5’ Attachment** | **3’ Attachment** |
| --- | --- | --- | --- | --- | --- | --- |
| AcpP-RXR (5’) | *acpP* | CTC ATA CCT TG | CAA GGT ATG AG | -6 to +5 | (RXR)_4_XB | H |
| AcpP-RXR (3’) | *acpP* |  |  |  | TEG | (RXR)_4_XB |
| AcpP-R_6_G (3’) | *acpP* |  |  |  | TEG | R_6_G |
| LpxC-RXR (5’) | *lpxC* | GTT GTT TGA TC | GAT CAA ACA AC | +3 to+13 | (RXR)_4_XB | H |
| LpxC-RXR (3’) | *lpxC* |  |  |  | TEG | (RXR)_4_XB |
| RpsJ-RXR (5’) | *rpsJ* | CCT CAG ACT CC | GGA GTC TGA GG | -15 to -5 | (RXR)_4_XB | H |
| RpsJ-RXR (3’) | *rpsJ* |  |  |  | TEG | (RXR)_4_XB |
| RpsJ-R_6_G (3’) | *rpsJ* |  |  |  | TEG | R6G |
| Scr- RXR (5’) | none | TCT CAG ATG GT |  | N/A | (RXR)_4_XB | H |
| Scr-RXR (3’) | none |  |  |  | TEG | (RXR)_4_XB |
| Scr-R_6_G (3’) | none |  |  |  | TEG | R_6_G |

**Table S.2:** Lead PPMOs used. Compounds are listed in alphabetical order. For the location relative to the start site we defined ‘A’ of ATG as +1. Abbreviations are as follows: R, (arginine); G (Glycine); X, 6-aminohexanoic acid (aminocaproic acid); B: ß-alanine (for conjugation); TEG: triethylene glycol; Scr: scrambled PPMO.
